# Effect of Adhesive Resin Application on the Durability of Adhesion to CAD/CAM Glass-Ceramics after either Hydrofluoric Acid Etching or Self-etch Primer Application

**DOI:** 10.3290/j.jad.b3240691

**Published:** 2022-08-18

**Authors:** Taciana Emília Leite Vila-Nova, Dayanne Monielle Duarte Moura, Gabriela Monteiro de Araújo, Rafael de Almeida Spinelli Pinto, Fabíola Pessôa Pereira Leite, Renata Marques Melo, Mutlu Özcan, Rodrigo Othávio de Assunção e Souza

**Affiliations:** a PhD Student, School of Dentistry, University of Pernambuco, Recife, PE, Brazil. Hypothesis, performed the experiments, and wrote manuscript.; b Professor, State University of Rio Grande do Norte (UERN), Department of Dentistry, Caicó, RN, Brazil. hypothesis, performed the experiments, and wrote manuscript.; c PhD Student, Federal University of Rio Grande do Norte (UFRN), Department of Dentistry, Division of Prosthodontics, Natal/RN, Brazil. hypothesis, performed the experiments, and wrote manuscript.; d Dentist, Federal University of Juiz de Fora (UFJF), Department of Restorative Dentistry Juiz de For a, MG, Brazil. Performed thermocycling.; e Professor, Federal University of Juiz de Fora (UFJF), Department of Restorative Dentistry. Juiz de Fora, MG, Brazil. Performed thermocycling.; f Researcher, Department of Dental Materials and Prosthodontics, Institute of science and technology, University Estadual Paulista- UNESP, São José dos Campos, SP, Brazil. Proofread the manuscript.; g Professor, University of Zurich, Center for Dental Medicine, Division of Dental Biomaterials, Clinic for Reconstructive Dentistry, Zurich, Switzerland. Analysis, discussion, revision.; h Adjunct Professor, Federal University of Rio Grande do Norte (UFRN), Department of Dentistry, Division of Prosthodontics, Natal, RN, Brazil. Idea, consulted, evaluated the results, statistical analysis.

**Keywords:** adhesion, glass-ceramic, shear bond, treatment surface, bonding, CAD/CAM, dental ceramics, dental materials.

## Abstract

**Purpose:**

To evaluate the effect of two surface conditioning methods, namely conventional hydrofluoric acid vs self-etching primer, and the application of adhesive on the bond strength of composite cement to CAD/CAM glass-ceramics.

**Materials and Methods:**

Blocks (N = 96) (12 x 10 x 2.5 mm) were manufactured, 24 for each tested ceramic type: lithium silicate ceramic (LS), polymer-infiltrated ceramic (PIC), leucite-reinforced feldspathic ceramic (FD), and lithium-disilicate glass-ceramic (LD). For bond strength testing, 64 blocks were randomly divided into 16 groups (4 blocks per group) according to the following factors: ceramic: 4 levels; etching: 2 levels (HFS: hydrofluoric acid + silane or Monobond Etch & Prime [MEP]); and adhesive application: 2 levels, with (signified as A) and without. Then for each group, 15 composite cement cylinders (AllCem Dual, FGM) were built up. All specimens were subjected to thermocycling (10,000 cycles) and to shear bonding strength testing (SBS) (100 kgf, 0.5 mm/min). Mean shear stresses (MPa) were statistically analyzed by three-way ANOVA, Tukey’s test, and Weibull analysis.

**Results:**

The mean bond strength of group PIC-HFS-A (28.45 ± 7.6 MPa) was significantly higher than that of groups LS-HFS-A (12.11 ± 2.7 MPa) and FDHFSA (20.86 ± 2.0 MPa). Group PIC-HFS bond strength (25.02 ± 6.5 MPa) was significantly higher only when compared to group LS-HFS (15.82 ± 4.4 MPa). The LS group presented lower SBS compared to all other groups. No significant differences were found between HFS and MEP surface treatments.

**Conclusion:**

Surface treatment with MEP promotes adhesion similar to that of HFS. Additional application of adhesive after the surface treatments did not improve the bond strength.

Although current glass-ceramics present different chemical compositions, several studies on adhesion have recommended that the surface treatment of these ceramics should be solely conditioning with hydrofluoric acid, followed by silanization, as it provides higher shear bond strengths.^3,9,11^ Also, some studies and manufacturers have suggested variations in the clinical adhesive cementation protocol, which includes adhesives after silanization, indicating that this additional step can mechanically and adhesively improve the restoration.^8,40^ Nevertheless, resin components of the adhesive should strengthen the ceramic by filling the cracks and surface irregularities that were previously induced by hydrofluoric acid, reducing the risk of failures. However, this procedure is still controversial.^11,16,17^

In this context, in order to simplify this clinical stage and optimize the adhesion between glass-ceramics and composite cements, another conditioning strategy has been introduced: a self-etching glass-ceramic primer. The self-etching glass-ceramic primer^18,19^ simultaneously provides conditioning and silanization of the ceramic surface, simplifying the steps prior to cementation. This treatment has the advantage of eliminating separate acid conditioning and silane application, besides being a faster, simplified and single-step technique. According to the literature, Monobond Etch & Prime (Ivoclar Vivadent; Schaan, Liechtenstein) produces a chemically active surface, rich in silica, similar to that created by hydrofluoric acid etching; this uniform silane layer is less prone to hydrolytic degradation and more stable in the long run.^19^

Apart from the technical data provided by the manufacturer (Ivoclar Vivadent), there are still only a few studies on Monobond Etch & Prime which evaluated its effect on the adhesion of composite cements to different types of ceramics, mainly to relatively new glass-ceramics, such as polymer-infiltrated ceramics (PIC)^27,29^ and lithium silicate glass-ceramic (LS, Vita Suprinity). In addition to these aspects, most studies have used water storage as an aging strategy.^18,19,27,29,30^ Few studies have also investigated the effect of long-term aging using thermocycling,^31-33^ which can be an important predictor of the clinical performance of the adhesion of PIC and LS.^33^

This study aims to compare the effect of surface treatments with a conventional (HF and silane) vs a self-etching primer, and to verify the effect of adhesive application on the bond strength of composite cement to different types of CAD/CAM glass-ceramics. The null hypothesis was that none of the variables tested (surface treatment, adhesive application, or the type of ceramic) would affect the bond strength between composite cement and glass-ceramics.

## MATERIALS AND METHODS

**Fig 1:**
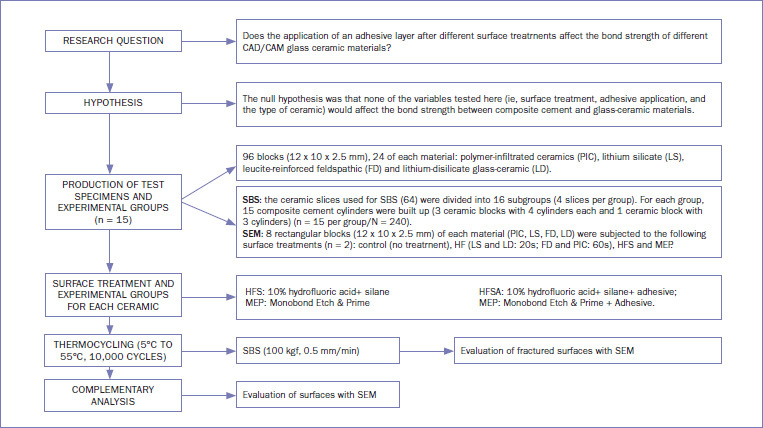
Flowchart of the study protocol. SBS: shear bond strength.

Material types, brands, manufacturers, chemical compositions, and batch number of the materials used in this study are presented in Table 1. The flowchart of the study design is shown in Fig 1.

**Table 1:** Materials, brands, manufacturers, composition, and batch numbers of all materials used in the present study

Material	Trade name	Manufacturer	Composition	Batch
PIC Polymer-infiltrated ceramics	Vita Enamic	VITA Zahnfabrik; Bad Säckingen, Germany	86% ceramic (58–63% SiO_2_, 20–23% Al_2_O_3_, 9–11% Na_2_O, 4–6% K_2_O, 0–1% ZrO_2_) 14% polymer (UDMA, TEG-DMA)	49601
LS Lithium silicate ceramic	Vita Suprinity		56–64% SiO_2_, 1–4% Al_2_0_3_, 15-21% Li_2_O, 8–12% ZrO_2_, 1–4% K_2_O	51523
Feldspathic ceramic	IPS Empress CAD	Ivoclar Vivadent; Schaan, Liechtenstein	SiO_2_, Al_2_O_3_, K_2_O, Na_2_O	T28994
Lithium-disilicate glass-ceramic	IPS e.max CAD		SiO_2_, Li_2_O, K_2_O, MgO, Al_2_O_3_, P_2_O	T38577
Self-etch ceramic primer	Monobond Etch & Prime		Ammonium polyfluoride, trimethoxypropyl methacrylate, alcohols, water	V09353
Hydrofluoric acid	Condac Porcelain		10% hydrofluoric acid	131217
Silane	PROSIL	FGM; Joinville, SC, Brazil	3-methacryloxypropyltrimethoxysilane, ethanol, water	290817
Conventional adhesive	ÂMBAR		UDMA, HEMA methacrylate acid monomers, methacrylate hydrophilic monomers, ethanol, water, silica nanoparticles, photoinitiators, co-initiators, stabilizers	–
Dual-cure composite cement	ALL CEM		Bis-GMA, bis-EMA, TEG-DMA, co-initiators, Initiators (camphorquinone and dibenzoyl peroxide), stabilizers, barium-silicate glass microparticles, and silicon dioxide nanoparticles	230517


### Preparation of Samples

CAD/CAM blocks (15 x 12 x 10 mm) of four ceramic restorative materials – lithium silicate: LS (Vita Suprinity, VITA Zahnfabrik; Bad Säckingen, Germany); polymer-infiltrated ceramic: PIC (Vita Enamic, VITA Zahnfabrik); leucite-reinforced feldspathic ceramic: FD (IPS Empress CAD, Ivoclar Vivadent; Schaan, Liechtenstein); and lithium-disilicate glass-ceramic: LD (IPS e.max CAD, Ivoclar Vivadent) – were sectioned using a double-sided diamond disk (22 x 0.15 mm, Dhpro; Paraná, Brazil) mounted on a straight micromotor handpiece under air-water spray, resulting in 24 rectangular slices per material with dimensions of 12 x 10 x 2.5 mm^3^, and subsequently measured with a digital caliper (Eccofer; Curitiba, Paraná, Brazil).^32^ One of the surfaces was polished in a polishing machine (Labpol 8-12, Extec; Enfield, CT, USA) using silicon carbide sandpaper (grit size: #200, #400, #600, #800, and #1200, 3M Oral Care; St Paul, MN, USA) under constant water cooling. Slices were then subjected to a digital ultrasonic cleaner for 5 min using distilled water. Afterwards, the LS and LD slices were sintered according to the manufacturer’s recommendations. The other materials do not require sintering. Sixty-four ceramic slices (16 per material) were used in shear bond strength (SBS) testing, and 32 slices (8 of each material) were utilized for SEM observation.

### Embedding of Ceramic Blocks

To test the SBS, all 64 ceramic slices were embedded in chemically activated acrylic resin (JET, Classic Dental Articles; São Paulo, SP, Brazil) using silicone molds (Master-Talmax silicone; Curitiba, Paraná, Brazil). After resin polymerization, the ceramic surfaces were polished with SiC sandpaper of increasing grit size (#600, #800, and #1200) on a polishing machine (Labpol 8-12, Extec) until acrylic resin excess was removed. Then, the total number of ceramic slices used for SBS (64) was divided into 16 subgroups (4 slices per group): ceramic: 4 levels (LS, PIC, FD, LD); etching treatment: 2 levels (hydrofluoric acid + silane [HFS] or Monobond Etch & Prime [MEP]); adhesive application: 2 levels (with [A] and without) as shown in Fig 1.^49^ For groups with the adhesive, a conventional adhesive (Ambar, FGM; Joinville, SC, Brazil) was applied according to the manufacturer’s instructions.

### Surface Treatments

Prior to surface treatment procedures, all ceramic slices were immersed in distilled water and cleaned in an ultrasonic device for 5 min (Cristófoli Equipment of Biosecurity; Paraná, Brazil), then left to dry for about 10 min. Subsequently, the adhesive area was delimited by a piece of adhesive tape (Scotch tape, 3M; Ribeirao Preto, Brazil) with a 3-mm-diameter perforation. Surface treatments were performed by one operator, according to the respective group:

- HFS groups: When adhesive application was omitted (HFS group), the ceramic block surface was etched with 10% HF (Condac, FGM) for the time indicated by each ceramic’s manufacturer; 60 s for FD and PIC, and 20 s for LS and LD. After the blocks were washed with air-water spray and air dried, a layer of silane (Prosil, FGM) was applied using a microbrush (Dentsply Sirona; Konstanz, Germany) and left for 2 min to allow the solvent to evaporate, according to the manufacturer’s recommendations. When adhesive was applied (HFSA group), the ceramic block surface was conditioned with HF and silane, as described above. After silanization, a layer of conventional adhesive (Ambar, FGM) was rubbed in using a microbrush for 10 s, then a second layer was applied for another 10 s. Excess adhesive was removed with the help of a microbrush, dried with an air jet for 10 s, and photoactivated for 10 s with an LED curing light (1200 mW/cm^2^, Radii Cal, SDI; Bayswater, Victoria, Australia), regularly measuring the output using a radiometer.
- MEP groups: For groups without adhesive application, a layer of self-etching ceramic primer (Monobond Etch & Prime [MEP]) was applied to the ceramic surface with the aid of a microbrush and rubbed for 20 s. After a reaction time of 40 s, the product was removed with air-water spray for 10 s and the surface was left to air dry. For groups with adhesive application, the surface was treated as described above. After washing and drying the MEP, the conventional adhesive was applied as described above.

### Adhesive Cementation and Thermocycling

After surface treatment procedures, 15 composite cement cylinders per group were built up (3 ceramic blocks with 4 cylinders each and 1 ceramic block with 3 cylinders) (N = 15 per group/N = 240).^48^ For this, a Teflon matrix (Ø = 2 mm: h = 2 mm) (Ultradent Jig, Ultradent; South Jordan, UT, USA) was used. After adapting the matrix, the composite cement base and catalyst paste were mixed for 10 s, inserted into the matrices with applicator tips, and then photoactivated for 40 s. After 5 min of chemical curing (as indicated by the manufacturer), the matrices were removed and the specimens (block + composite cement cylinder) were subjected to thermocycling (10,000 cycles) in alternating baths of 5ºC and 55ºC with 30-s dwell time and 5-s transfer time (Nova Etica; Sao Paulo, Brazil).

### Shear Bond Strength (SBS) Test

After thermocycling, the composite cement cylinders were subjected to shear bond strength testing on a universal testing machine (Instron 3365; Norwood, MA, USA) carried out by one operator. The block-cylinder assemblies were fixed into a metal device to allow the block-cement interface to remain perpendicular to the horizontal plane. The upper portion of the device, resembling a metal chisel, touched the adhesive interface area and generated shear stresses at a speed of 0.5 mm/min with a 100-kgf load until specimen fracture. The adhesive strength was calculated by the formula R = F/A, where R = adhesive strength (MPa), F = force (N), A = interfacial area (area of a circle in mm). The adhesive area of each composite cement cylinder was defined as the area of a circle, calculated as A = π^2^, where π = 3.14, and r = 1 mm.

### Failure Type Analysis

The fractured surfaces were examined using an optical stereomicroscope (Stereo Discovery V20, Zeiss; Göttingen, Germany) and the failure mode of representative specimens was analyzed using SEM (Inspect S50, FEI; Brno, Czech Republic). The failure modes were defined as follows:^55^ A: adhesive of ceramic/cement interface; B: cohesive within ceramic; C: cohesive within cement; D: mixed 1, predominantly adhesive failure of ceramic/cement interface + cohesive cement failure; E: mixed 2, adhesive failure of ceramic-cement interface + ceramic cohesive failure.

### Scanning Electron Microscopy (SEM)

Two representative samples from each group (with 8 ceramic slices of each material: LS, PIC, FD, LD) were prepared as previously described, and further subjected to the following surface treatments (N = 2): control (no treatment), HF (according to the respective manufacturer’s recommendations: LS and LD for 20 s; FD and PIC for 60 s), HFS and MEP. The specimens were first assessed in an optical stereomicroscope and then analyzed using SEM at 2500X magnification (HITACHI, model TM 3000, Hitachi; Tokyo, Japan).

### Statistical Analysis

The statistical n considered in statistical analysis was the number of resin cylinders subjected to shear bond strength testing (N = 15/per group). According to their groups, the mean SBSs were obtained from the means of all the composite cement cylinders. Power was calculated through the website www.openepi.com by comparing the maximum and minimum means ± SD of SBS data, considering a 95% confidence interval and a sample size of 15 per group. Three-way ANOVA and Tukey’s test were used to compare data between groups using Statistix software (version 8.0, 2003, Analytical Software; Tallahasee, FL, USA). The level of significance was set at 5%. Failure mode and SEM results underwent statistical descriptive analysis.

Weibull modulus (m) and characteristic strength (σ_0_) were obtained using the Weibull analysis to evaluate the reliability of the shear bond strength data considering strength variation. Characteristic strength is the strength at a failure probability of approximately 63.3%. Weibull modulus and characteristic strength with a 95% confidence interval were calculated as follows: ln{ln [1/(1 – F(σ_c_)]} vs lnσ_c_ (according to ENV 843-5):


$$\ln\ln\left(\frac{1}{1-F\left(\sigma_{c}\right)}\right)=m\ln\sigma_{c}-m\ln\sigma_{o}$$

Weibull analysis was performed using Minitab software (version 17, 2013, Minitab; State College, PA, USA). The level of significance was set at 5%.

## RESULTS

### Shear Bond Strength (SBS) and Weilbull Distribution

Considering a 95% confidence interval, the power of the SBS data was 100%. Three-way ANOVA revealed that the factor “ceramic” (p = 0.0000) and the interaction “ceramic x etching treatment” (p = 0.0003) were significant. However, the factors “adhesive application” (p = 0.237) and “etching treatment” (p = 0.605) and their interactions did not influence the results.

When all experimental groups were analyzed, Tukey’s test showed that for the groups with adhesive application (HFS-A), mean bond strength in the group PIC-HFS-A (28.5 ± 7.6 MPa) was significantly higher than in groups LS-HFS-A (12.1 ± 2.7 MPa) and FD-HFS-A (20.9 ± 2.0 MPa). For etching treatment without adhesive application (HFS), the PIC-HFS specimens (25.0 ± 6.5 MPa) were significantly different only when compared to LS-HFS (15.8 ± 4.4 MPa). For the MEP groups treated with and without the adhesive, the LS group presented significantly lower SBS when compared to all other groups (Tukey’s test p < 0.05). Also, the SBS results showed no significant differences between HFS and MEP for any of the ceramic types (p = 0.20). The SBS means ± SD of experimental groups are shown in Table 2.

**Table 2:** Shear bond strengths (means ± SD) in MPa of ceramic types submitted to different etching treatments (HFS and MEP) and adhesive application

Etching treatment	Adhesive aplication	Ceramic type			
		PIC	LS	FD	LD
HFS	Yes	28.45 ± 7.6^Aa^	12.11 ± 2.7^Ca^	20.86 ± 2.0^Ba^	25.25 ± 7.7^ABa^
	No	25.02 ± 6.5^Aa^	15.82 ± 4.4^Ba^	20.95 ± 5.5^ABa^	24.21 ± 3.1^Aa^
MEP	Yes	22.09 ± 6.7^Aa^	12.62 ± 3.4^Ba^	24.04 ± 7.8^Aa^	25.30 ± 6.0^Aa^
	No	21.62 ± 7.0^Aa^	11.20 ± 3.1^Ba^	25.98 ± 7.1^Aa^	22.85 ± 5.0^Aa^

Superscript uppercase letters indicate statistically significant differences between different ceramic types for the same etching treatment and adhesive application (p < 0.05); superscript lowercase letters indicate statistically significant differences between ceramic types within the same etching treatment with and without adhesive application (p < 0.05). HFS: hydrofluoridric acid + silane; MEP: Monobond Etch & Prime; PIC: polymer-infiltrated ceramic (Vita Enamic); LS: lithium silicate ceramic (Vita Suprinity); FD: leucite-reinforced feldspathic ceramic (IPS Empress CAD); LD: lithium-disilicate glass-ceramic (IPS e.max CAD).

**Fig 2:**
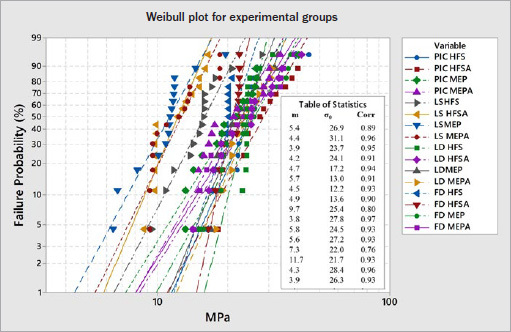
Weibull plot of the shear bond strengths of the experimental groups.

The Weibull modulus (m) and characteristic strength (σ_0_) of the experimental groups were statistically different from each other (p = 0.00). The groups of LS ceramic type presented significantly lower characteristic strength than the other groups. The Weibull distributions are graphically shown in Fig 2, and associated parameters are summarized in Table 3.

**Table 3:** Weibull modulus (m), characteristic strength (σ_0_) and respective CI (95%) of SBS by surface treatment and ceramic material

Groups	m	Cl	σ_0_	Cl
PIC HFS	5.4^ABC^	4.4–6.6	26.9^ab^	24.2– 29.9
PIC MEP	3.9^C^	2.9–5.1	23.7 ^abc^	20.6–27.3
PIC HFSA	4.4^ABC^	3–6.4	31 ^a^	27.5– 35
PIC MEPA	4.2^ABC^	3–5.9	24 ^ab^	21.1–27.4
LS HFS	4.7^BC^	3.7–6	17.2 ^c^	15.3–19.3
LS MEP	4.5^BC^	3.4–5.8	12.2^d^	10.8–13.8
LS HFSA	5.7^ABC^	4–8.1	13 ^d^	11.8–14.3
LS MEPA	4.9^ABC^	3.7–6.6	13.6 ^d^	12.2–15.2
FD HFS	7.3^AB^	6.3–8.3	22 ^bc^	20.2–24.1
FD MEP	4.3^ABC^	3–6.3	28.4^a^	25.1–32.2
FD HFSA	11.7^ABC^	6.1–22.4	21.7^bc^	20.8–22.7
FD MEPA	3.9^BC^	2.8–5.4	26.3^ab^	22.9–30.3
LD HFS	9.7^A^	7.3–13.1	25.3 ^ab^	24–26.8
LD MEP	5.8^ABC^	4.3–7.8	24.5 ^ab^	22.3–27
LD HFSA	3.8^BC^	2.7–5.5	27.8 ^ab^	24.1–32
LD MEPA	5.6^ABC^	4.4–7.1	27.2 ^as^	24.6–30

Same superscript uppercase letters indicate statistical similarity among Weibull moduli. Same superscript lowercase letters indicate statistical similarity among Weibull characteristic strengths (p < 0.05).

### Fracture Surface Analysis

**Fig 3:**
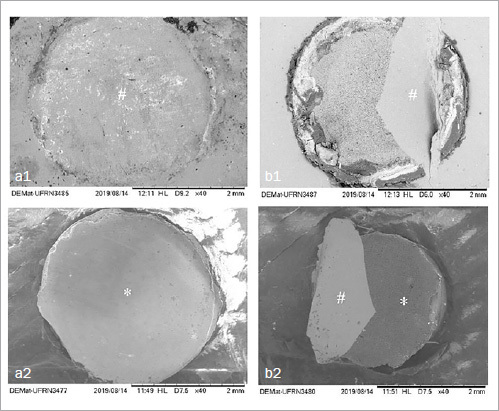
SEM images (40X) showing failure types at the interface. *cement; #ceramic. a: adhesive failure at the ceramic/ composite-cement interface. a1: ceramic; a2: cement cylinder preserved due to adhesive failure); b: mixed adhesive failure type 2: ceramic/cement interface and cohesive in ceramic: b1: ceramic; b2: cement cylinder with remnants of cohesively failed ceramics.

The predominant failure mode for all materials was mixed. For LS ceramic, the presence of mixed failure type 1 was predominant for all groups. Regarding PIC, the percentage of mixed failure types 1 and 2 were equivalent, except for the MEP group, which had a higher percentage of adhesive failures (40%). For FD, mixed failure type 2 was predominant for the groups with MEP (93%) and MEP+A (86%), and in the groups receiving HF surface treatment, mixed failure type 1 (73%) was more frequent. Finally, for LD, type 1 mixed failures were most common. Also for this material, the MEP group presented a higher percentage of adhesive failures (54%). The complete results for failure mode analysis are shown in Table 4. Representative images of failure modes are shown in Fig 3.

**Table 4:** Failure analysis of each ceramic material per surface treatment and pre-test failures during thermocycling

Material	Groups	Failure type						
		Cement/ ceramic adhesive	Cohesive in cement	Cohesive in ceramic	Mixed 1: adhesive cement/ ceramic/ cohesive cement	Mixed 2: adhesive cement/ ceramic/ cohesive ceramic	Pre-test failure	Total
PIC	HFS	–	–	–	9 (60%)	6 (40%)	–	15 (100%)
	MEP	6 (40%)	–	–	2 (14%)	7 (46%)	–	15 (100%)
	HFSA	–	1 (7%)	–	8 (55%)	6 (60%)	–	15 (100%)
	MEPA	2 (14%)	–	–	6 (40%)	7 (46%)	–	15 (100%)
LS	HFS	3 (20%)	–	–	11 (73%)	1 (7%)	–	15 (100%)
	MEP	4 (27%)		–	9 (60%)	2 (13%)	–	15 (100%)
	HFSA	–	1 (7%)	–	13 (86%)	1 (7%)	–	15 (100%)
	MEPA	–	–	–	13 (86%)	2 (14%)	–	15 (100%)
FD	HFS	1 (7%)	–	–	11 (73%)	3 (20%)	–	15 (100%)
	MEP	–	–	–	1 (7%)	14 (93%)	–	15 (100%)
	HFSA	1 (7%)		–	11 (73%)	3 (20%)	–	15 (100%)
	MEPA	–	–	–	2 (14%)	13 (86%)	–	15 (100%)
LD	HFS	1 (7%)	–	–	12 (79%)	2 (14%)	–	15 (100%)
	MEP	8 (54%)	–	–	7 (46%)	–		15 (100%)
	HFSA	2 (14%)	–	–	11 (72%)	2 (14%)	–	15 (100%)
	MEPA	3 (20%)	–	–	10 (66%)	2 (14%)		15 (100%)

PIC: polymer-infiltrated ceramics; LS: lithium silicate; FD: leucite-reinforced feldspathic; LD: lithium-disilicate glass-ceramic. HF: hydrofluoric acid; MEP: Monobond Etch & Prime; A: adhesive; S: silane.

### Surface Morphology Analysis

**Fig 4:**
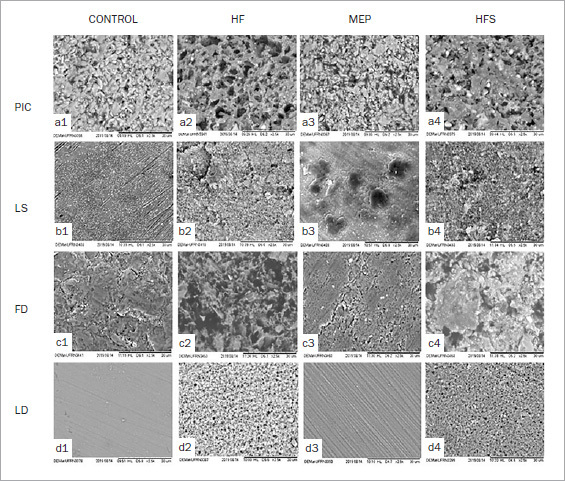
SEM images (1500X) showing different surface- treatment groups. a: polymer-infiltrated ceramics (PIC); b: lithium-silicate ceramic (LS); c: leucite-reinforced feldspathic ceramic (FD); d: lithium-disilicate glass-ceramic (LD). 1. control group (no treatment); 2. HF; 3. MEP self-etch ceramic primer; 4. HFS.

Representative SEM images of the materials with the different surface treatments are shown in Fig 4. For the LD, PIC and FD ceramics, HFS (Fig 4) resulted in surfaces with more irregularities and depressions compared to the untreated group and MEP groups, which presented a surface with considerably fewer irregularities. LS ceramic specimens presented surface roughness, but with fewer micropores.

## DISCUSSION

This study compared two etching treatments and the effect of applying a conventional adhesive after conventional etching with HFS or and a simplified (MEP) surface treatment on the bond strength of composite cement to different types of glass-ceramic. Four types of glass-ceramic were investigated in this study: lithium silicate ceramic (Vita Suprinity), polymer-infiltrated ceramic (Vita Enamic), leucite-reinforced feldspathic ceramic (IPS Empress CAD), and lithium-disilicate glass-ceramic (IPS e.max CAD). These materials vary in chemical composition, proportions of silica, and crystalline phases; however, they have similar clinical indications and adhesive characteristics.^59^ Based on the fact that the variable “type of ceramic” had a statistically significant effect on the SBS, the null hypothesis was rejected. The results showed that both after conditioning with HFS and after the application of MEP, LS ceramic showed significantly lower SBS, regardless of adhesive application or not, compared to the other ceramic types. Additionally, it was also observed that PIC, after HFS-A treatment, presented significantly higher SBS than did FD and LS ceramics.

LS is a glass-ceramic consisting of fine lithium metasilicate components with LD crystals and a glassy matrix containing 10% zirconium dioxide (zirconia, ZrO_2_).^4^ Although it has been reported that the presence of zirconia in LS ceramic can decrease its sensitivity to HF, several studies have reported that conditioning with HF followed by silane is the most reliable approach for this type of material.^3,45^ However, previous studies have suggested that LS ceramic (Vita Suprinity) has a different microstructure than other glass-ceramics, mainly LD glass-ceramic. Variations in the microstructure can produce different conditioning and roughness patterns when subjected to the same surface treatment.^10^ LS is a silica-based ceramic with an average crystal size of approximately 0.5 µm, whilst the LD glass-ceramic type (IPS e.max CAD) has a crystal with an average size of 1.5 µm.^10^ Additionally, LS ceramics have a lower concentration of silicon dioxide (56.0-64.0 µm) and a lower crystalline phase compared to the LD ceramics.^22^ Given that the mechanism of action of HF consists in a reaction in which the acid selectively dissolves the glassy phase, exposing the crystalline structure, changing the surface topography by making the surface porous, thus favoring micromechanical retention,^51^ this finer microstructure of LS can result in less solubility of the vitreous matrix and cause less roughness,^22^ explaining the lower SBS found in this study. Further, representative SEM images of this study illustrated that for LS, the conditioning pattern of the glassy matrix with HF and MEP did not reflect homogeneous dissolution of the vitreous phase and did not result in a conditioning pattern with microcavities and protrusion of the crystals as noted in LD, FD, and PIC ceramics.

Regarding PIC, the PIC-HFS-A group presented significantly greater SBS and characteristic strength (σ_0_) than did the FD-HFS-A group. The composition of PIC is an important factor in the conditioning of the surface, since it has a phase of feldspathic ceramic reinforced by leucite interconnected with a polymer. Similar results were reported in another study,^5^ in which PIC and FD ceramics were conditioned with 10% HF. It was observed that the contact angle of PIC surfaces was higher than that of FD.^5^ Some authors state that the PIC surface becomes more hydrophobic after etching, since it exposes the infiltrated polymer.^5,25,53^ This increases adhesion, especially with the adhesive, allowing enhanced chemical interaction of the adhesive with the polymer in the infiltrated ceramic.^28^ Conditioning with HF removes part of the glassy matrix and part of the polymer, producing microporosities and microchannels, as also demonstrated by representative SEM images from our study (Fig 4, a2). As the conditioning pattern with MEP is more superficial, this surface treatment seems to be a better option for this material.^13^ However, the two surface treatments proved to be the same in terms of bonding. Thus, further studies should be carried out to confirm this interaction.

In terms of SBS, was no significant difference was found between HFS and MEP for any of the ceramic types, which demonstrates that both surface treatments can be used for the four materials tested here. Several studies have reported that the application of HF followed by silane is still the preferred protocol for the surface treatment of ceramic restorations, because it offers high SBS to composite cements and long-term adhesion.^6,18,32,56^ On the other hand, simplified techniques have also been shown to be a clinically viable option,^49^ especially because they do not pose the danger of toxicity through the use of HF.^26,40^ Moreover, the conventional HFS technique requires a sequence of clinical steps, wherease MEP reduces to a single step conditioning and silanization.^23^ This decreases the probability of errors in the cementation protocol, provides greater control of ceramic exposure to the acid solution, while avoiding deleterious effects on the flexural strength of glass-ceramics which often result from increased surface porosity caused by HF if applied too long or at excessive concentrations.^57^

Corroborating the results of this study, other authors have reported similar results comparing MEP and HFS for bonding composite cement to glass-ceramics. MEP involves active application of the silane contained in MEP, promoting greater hydrophobicity on the ceramic surface and enhancing its interaction with composite cement.^52^ Nonetheless, these results might be due to MEP’s reaction mechanism, which is not yet entirely clear.^8^ This single-step self-etching primer is applied to the ceramic surface and rubbed in for 20 s, removing saliva and silicone oils, guaranteeing a clean surface. Thereafter, for 40 s, ammonium polyfluoride acid reacts with the ceramic, creating a chemically active, rougher surface, on which the silane component of MEP binds to silicon oxides. After this reaction time, the ceramic surface is washed with air-water spray, removing the acidic solution, and then air dried until moisture is not longer visible. Subsequently, the silane condenses on the ceramic surface and forms a stable and chemically reactive layer for methacrylate end groups, providing high chemical adhesion to composite cement.^23,30,31^

Similar to our findings, stable adhesion of MEP has been reported by clinical^36^ and in vitro^9,52^ studies, showing that even after thermocycling, this surface treatment offers SBS similar to conventional treatment. However, other authors have reported that the success of MEP’s mechanism of action is directly related to the stability of silane molecules in acidic environments.^8,58^ It is known that the continuous hydrolysis of silanol groups occurs during long-term storage in a low pH solution. Although the manufacturer reports that silane is stable during storage in ammonium polyfluoride, the condensation reaction of the silanol groups is suppressed.^8,23^ The formation of a highly reactive monomeric silanol is continuously promoted during storage.^8,23^ Hence, in the presence of this chemical reaction, the MEP mechanism becomes based on an interaction between functional phosphoric monomers and ceramic ions instead of methacrylate bonding of silane to the glass-ceramic; this interaction needs further study.^8^ In general, MEP demonstrated similar results to HFS in this study, and may be a good clinical option in the surface treatment of glass-ceramics.^17,42,51^

Regarding the additional adhesive application treatment, the product used was a conventional adhesive indicated for the cementation of glass-ceramic restorations (manufacturer’s information). Our results showed groups with adhesive application performed as well as groups without adhesive for all materials. Agreeing with our data, most authors report that the use of adhesive after silane is not necessary and can be omitted from the workflow.^16,40,41,43^ This additional clinical step may increase the possibility of errors and generate losses in long-term adhesion. The latter results from the fact that when the silane/adhesive/cement interfaces are subjected to aging in water, hydrolytic degradation of the adhesive layer occurs more quickly due to its water absorption.^16,40^ Souza et al^48^ investigated the application of adhesive after silanization of vitreous ceramics with HFS and with MEP, finding that the use of an adhesive after the surface treatments did not siginificantly improve adhesion,^48^ just as in the present study. Additionally, in a recent systematic review, the authors also reported that the additional application of adhesive after silanization did not significantly improve the SBS and could be an unnecessary step.^36^

Moreover, the use of adhesives after silane increases the likelihood of chemical incompatibility between some adhesives and composite cement, compromising the adhesion even more.^46^ However, other authors have reported that chemical incompatibility between conventional adhesives and dual-curing composite cement was not important.^20^ Based on our results and those of other studies, we do not recommend adhesive application after silanization of the ceramic surface.^36^ Thus, the surface treatment of ceramic restorations can be successfully executed using HFS or MEP without applying adhesive, and several types of cementation systems (eg, self-etching, conventional, or self-adhesive) can be employed.^36^ However, before cementation, the manufacturer’s recommendations must be followed to avoid problems at the composite cement/dental substrate interface.

The analysis of failure modes (Table 4) showed that most of the failures were adhesive or type-1 mixed mode with a predominance of adhesive failures. The frequent adhesive failures may indicate lower adhesion of the composite cement to the ceramic surface.^13^ Although adhesive failures were common at the cement-ceramic interface, this did not affect adhesion to the composite cement for FD, PIC, and LD ceramics, which was not the case for LS ceramic, as shown by the SBS results in our study. For the MEP groups in the PIC and FD groups, the presence of type-2 mixed failure may indicate that the surface treatment applied promoted a bond so strong that the testing caused the ceramic material to fail cohesively. However, adhesive failure was still prevalent, which did not prove to be significant in the SBS test results.^55^ Associated with this, although the shear bond test is used to assess the adhesion level, it is known that the non-uniform distribution of stresses during the shear movement can also result in cohesive failures in both composite cement and ceramic.^37^

Thus, the Weibull analysis confirmed the results obtained in the SBS test. Although the Weibull modulus is relatively low for bond strength data, it can be used in combination with the characteristic strength (σ_0_), as an indicator for choosing materials or techniques.^31^ Our Weibull analysis results showed that the surface treatment with HFS and MEP showed statistically similar values of (m) for all ceramic types. However, the characteristic strength (σ_0_) results for LS ceramic were statistically lower for all groups and indicated the lower structural reliability of these adhesive interfaces. This confirmed the results obtained for the SBS test. For FD ceramic treated with MEP, the σ_0_ was significantly higher than that of the HFS group, which would indicate higher structural reliability of these adhesive interfaces and a lower probability of fractures.^7^ For all ceramic types, the m and σ_0_ also demonstrated no significant differences between the groups with and without adhesive, which reinforces the conclusion that additional adhesive application did not offer higher reliability.

All specimens in this study were subjected to 10,000 thermocycles, which simulates conditions equivalent to one year of clinical use.^21,47^ This process promotes faster hydrolytic degradation of the interface due to its contraction and expansion stresses as a consequence of different thermal expansion coefficients among different materials, which is considered an important predictor of the adhesive performance of restorative interfaces.^35^ Further studies evaluating the effects of these surface treatments, especially for LS and PIC, are needed. Additional analyses, such as EDS (energy dispersive spectroscopy) and SEM interface examination, should be performed to support the results obtained here.

Future studies evaluating the different types of adhesive resin and their chemical interactions with MEP, including other cementation techniques, may further complement the results of this study.

## CONCLUSIONS

Conventional surface treatment with hydrofluoric acid followed by silane (HFS) or Monobond Etch & Prime (MEP) application did not show significant differences between the ceramic types tested. LS ceramic demonstrated lower SBS after HFS and MEP treatments when compared to FD, PIC and LD ceramics. Additional application of the adhesive resin after silanization or MEP did not improve the bond strength; thus, it can be omitted from the clinical workflow.

## Clinical relevance:

The self-etching ceramic primer Monobond Etch & Prime (Ivoclar Vivadent) seems to be a good option to simplify cementation of ceramic restorations, and an additional application of adhesive after surface treatment can be omitted from the cementation workflow for the ceramic materials tested.
